# Alcohol use disorder treatment and outcomes among hospitalized adults with alcoholic hepatitis

**DOI:** 10.1016/j.dadr.2021.100004

**Published:** 2021-11-17

**Authors:** Adam C. Winters, Folasade P. May, Yun Wang, Paul Shao, Liu Yang, Arpan A. Patel

**Affiliations:** a Vatche and Tamar Manoukian Division of Digestive Diseases, Department of Medicine, David Geffen School of Medicine at UCLA, Los Angeles, CA; b Veterans Affairs Greater Los Angeles Healthcare System, Los Angeles, CA; c Department of Medicine, David Geffen School of Medicine at UCLA, Los Angeles, CA

**Keywords:** Alcohol-use disorder, Alcohol-associated liver disease, Alcoholic hepatitis

## Abstract

**Purpose:**

**:** The burden of alcohol-associated liver disease (ALD) in the United States (US) has continued to worsen in the background of rising rates of alcohol use disorder. Patients with ALD present to care at a late stage, often with the sequela of liver decompensation, such as gastrointestinal bleeding and infection. ALD is now the leading indication for liver transplantation. We aimed to measure the quality of care delivered to hospitalized patients with alcoholic hepatitis (AH) across 3 domains: 1) alcohol-use disorder (AUD) care, 2) inpatient cirrhosis care, and 3) alcohol-associated liver disease (ALD) care—and observe associations between quality of care and outcomes.

**Methods:**

**:** We included hospital encounters between January 1, 2016 and January 1, 2019 to a large, diverse integrated health system for AH with active alcohol use within the prior 60 days. The diagnosis of AH was determined based on previously published clinical and laboratory criteria. Quality indicator (QI) pass rates were calculated as the proportion of patients eligible for each indicator who received the QI within the timeframe specified. We then evaluated the association between the receipt of all QIs and 6-month mortality, as well as AUD-specific QIs and 30-day readmission.

**Results:**

**:** Of the 179 patients, the median age was 47 years-old, 59.2% were male and 49.2% were non-Hispanic White. The median Model for End-Stage Liver Disease-Sodium score was 25, while the median discriminant function was 33. Patients were followed for an average of 21 months. Overall, 14% of patients died during the index hospitalization while 17.3% died following discharge and 24.8% were re-admitted within 30-days. QI pass-rates were variable across the different domains. Few patients received AUD care—pass rates for receipt of pharmacotherapy and behavioral therapy at 6 months were only 19.1% and 35.1%, respectively. There was a significant association between receiving behavioral therapy and 6-month mortality—3% vs 18%, *p* = 0.05.

**Conclusion:**

**:** The quality of care received during hospital encounters for AH is variable, and AUD-specific therapy is low. Future quality of care initiatives are warranted to link patients to AUD treatment to ensure optimal care and maximize patients survival in this at-risk population.

## Highlights

• Receipt of addiction-related care post-discharge for patients hospitalized with alcoholic hepatitis is low.
• Among a group of established quality indicators for patients with alcoholic hepatitis, only behavioral therapy for alcohol use disorder was associated with improved 6-month mortality.
• Linkage to AUD care in the immediate post-discharge setting is essential to ensuring optimal care in this vulnerable population.

## Introduction

The burden of alcohol-associated liver disease (ALD) in the United States (US) has continued to worsen in the background of a mounting public health crisis surrounding rising rates of alcohol use disorder (AUD). According to the 2019 National Survey on Drug Use and Health, nearly 15 million people age 12 and older have an AUD.(SAMHSA, 2019) Between the periods 2001–2002 and 2012–2013, the prevalence of AUD increased by 50% in the general population and by an alarming 84% in women. (Grant et al., 2017) Over a similar timeframe, ALD has become increasingly common and is now the leading indication for liver transplantation, supplanting hepatitis C infection.(Cholankeril and Ahmed, 2018) Similarly, annual healthcare costs attributed to alcohol-associated cirrhosis have outpaced costs related to other forms of liver disease.(Mellinger et al., 2018)

Hospitalizations represent an important opportunity for quality improvement in this population, since 1) hospitalizations from ALD (including alcoholic hepatitis [AH] and acute-on-chronic liver failure from alcohol use) are increasingly common(Cargill et al., 2020; Thompson et al., 2018), 2) inpatient stays are costly and morbid (Liangpunsakul, 2011; Thompson et al., 2018), and 3) future healthcare use, including mortality and readmissions, may very well be prevented. The standard of medical care provided to these patients in the setting, however, has grown increasingly complex. Patients with ALD present to care at a late stage (Shah et al., 2019), often with the sequela of hepatic decompensation, such as bleeding from esophageal varices and infection.(Yu et al., 2010) Medical teams may also need to consider liver transplantation for a subset of seriously ill patients and develop comprehensive plans for improving abstinence.

The first step to improving the quality of care delivered to this population is identifying quality gaps and their impact on healthcare use. In this study, we aimed to evaluate the receipt of evidence-based quality indicators (QIs) to actively drinking hospitalized patients with AH at a high-volume, academic tertiary healthcare system across 3 domains— treatment of comorbid alcohol use disorder, the inpatient management of complications of portal hypertension, and inpatient management of ALD—and observe associations between quality of care received for each domain and patient outcomes.

## Materials and methods

### Cohort identification

**Fig. 1:**
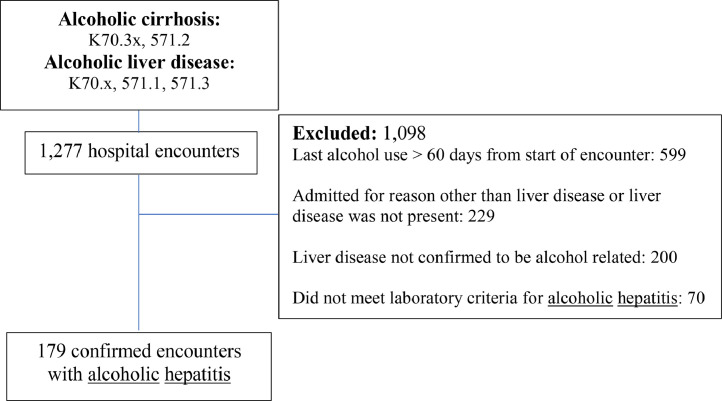
Study population with exclusion criteria.

We performed a retrospective chart review of hospital encounters for patients with AH who were admitted to UCLA Health (Ronald Reagan UCLA Medical Center or UCLA Medical Center, Santa Monica) between January 1, 2016 and January 1, 2019. We initially identified eligible hospital encounters using *International Classification of Diseases*, Ninth Revision (ICD‐9)(Nehra et al., 2013) or Tenth Revision (ICD‐10)(Mellinger et al., 2018) codes for “alcoholic cirrhosis” or “alcoholic liver disease” (See Fig. 1). Only care involving the patient's first hospitalization over the study period was assessed. Encounters were excluded if 1) the patient did not have liver disease, 2) the patient did not meet established criteria for AH (Crabb et al., 2016) on admission, or had a liver biopsy and/or clinical documentation from a gastroenterologist indicating the etiology of disease was not AH, or 3) last alcohol use was >60 days from the date of admission.

### Chart abstraction

Data were abstracted by a team of clinician abstractors with experience using the electronic health record (EHR). Each abstractor received training from research personnel on how to use a standardized data abstraction sheet to ensure consistency. Prior to data abstraction, each abstractor completed ten test cases and received further training if abstracted data did not match the test case solution set. Near the end of data abstraction, one primary clinician abstractor reviewed a 10% random sample of each abstractor's data sample to assure accuracy of data. The study team discussed and resolved any discrepancies by consensus.

### Patient characteristics

We abstracted EHR data for encounter demographics (age, gender, race, ethnicity, marital status, primary language, insurance status), admission laboratory studies (serum sodium, creatinine, total bilirubin, international normalized ratio), post-discharge visits to primary care (number of visits), and receipt of specialty care. If a patient was seen by a gastroenterologist or transplant hepatologist for ≥1 visit after discharge, he or she was classified as having received specialty care. Readmission within 30 days of discharge and 6-month mortality were measured for each encounter. We assessed outcomes through our own EHR, as well as that available from neighboring institutions through the Epic Care Everywhere function. (Ross et al., 2020)

### Quality indicators

We selected 9 different QI measures across 3 domains – 1) management of AUD, 2) management of hospitalized patients with cirrhosis, and 3) management specific to ALD. For the first domain, we selected two QIs focused on receipt of treatments for comorbid AUD among those who survived their hospitalization: receipt of 1) a prescription for pharmacotherapy for alcohol dependence and 2) a behavioral or psychosocial intervention, both within 6 months of discharge. These were selected from QIs developed for unhealthy alcohol use(Hepner et al., 2019), but the interval for receiving treatments was relaxed from 30 days to 180 days in order to align with a recent publication showing very low rates of receiving AUD treatment in ambulatory Veterans with ALD (Rogal et al., 2020). Pharmacotherapies we evaluated specifically were naltrexone, acamprosate, disulfiram, baclofen, gabapentin and topiramate. We counted the patient as having received behavioral therapy if there was documentation confirming a visit to addiction medicine or psychiatry—either addiction or general—for AUD care, attendance in an alcohol rehabilitation program, participation in counseling such as cognitive behavioral therapy or attendance of a 12-step group program.

For the second domain, we selected 4 QIs developed for inpatients with cirrhosis that were assigned the highest priority scores for standard of care in the inpatient setting (Kanwal et al., 2010): 3) patients with new or existing ascites admitted with symptoms related to ascites or hepatic encephalopathy should receive a diagnostic paracentesis, 4) patients with a polymorphonuclear leukocyte count ≥250 should receive antibiotics within 24 h, 5) patients admitted with or who developed gastrointestinal [GI] bleeding should receive antibiotics within 24 h, and 6) patients admitted or developed GI bleeding should undergo esophagogastroduodenoscopy [EGD] within 24 h. For the last domain, three QIs were selected based on the most recent American Association for the Study of Liver Disease guidance demonstrating practices of “proven” or “likely” benefit for patients with ALD(Crabb et al., 2020): 7) all patients should receive a nutrition consult, 8) patients meeting criteria for severe AH—defined as Model for End-Stage Liver Disease Score [MELD] >21(Dunn et al., 2005) or discriminant function [DF] >32—without an existing contraindication—should receive corticosteroids, and 9) all patients should receive counseling by the inpatient providers on abstinence from alcohol use, this was distinct from the post-discharge behavioral or psychosocial intervention evaluated in the AUD domain.

### QI adherence

For each encounter, we determined eligibility for each QI. We then determined the receipt of QIs either during the encounter or after discharge (for the AUD-related QIs). QI pass rates were calculated by dividing the number of patients that received the QIs by the number of patients eligible for that QI. Patients who did not have either primary care or specialty care follow up after discharge were not eligible for the two AUD QIs.

### Association between QI adherence and outcomes

We used Fisher's Exact Test to determine the association between the receipt of specific QIs and the study outcomes—6-month mortality for all QIs and 30-day readmission for the AUD QIs. We considered *P* ≤ 0.05 as statistically significant. We used SAS (version 9.4) for all statistical analyses. Due to the resultant small sample size, adjusted analyses were not performed.

## Results

### Demographics and clinical characteristics

There were 179 hospital encounters that met study inclusion and exclusion criteria over the study period (Fig. 1). Table 1 outlines the baseline characteristics of the cohort. Median age at the time of admission was 47 years (IQR 37–56). The cohort was mostly male (59%) and non-Hispanic White (49%). The study population had advanced disease as 111 (62%) met criteria for severe AH. The median admission MELD score was 25.2 (IQR 19.5–32.2) while median DF was 33 (IQR 14–56) and 109 (60.8%) had underlying cirrhosis. Post-encounter, patients were followed for a median of 21 months (IQR: 3–34). Fourteen percent died during the hospitalization, 17% died following discharge, and 21 (11.7%) underwent liver transplantation. Of the patients who survived their index hospitalization, 73% had at least one follow-up visit with a primary or specialty (GI and/or hepatology) care clinic, and 36 (24.8%) were re-admitted within 30 days.

**Table 1:** Cohort characteristics and outcomes.

**Variable**	***N*** **= 179**
**Age, median years (IQR)**	**47 (37–56)**
**Male Gender, N (%)**	**106 (59.2)**
**Race/Ethnicity, N (%)**	
**Non-Hispanic White**	**88 (49.2)**
**Non-Hispanic Black**	**11 (6.2)**
**Hispanic**	**52 (29.1)**
**Asian**	**6 (3.4)**
**Other/Unknown**	**22 (12.3)**
**Admission MELD-Na score, median (IQR)**	**25.2 (19.5–32.2)**
**Admission discriminant function, median (IQR)**	**33 (14–56)**
**Follow-up time, months (IQR)**	**21 (3–34)**
**Died during index hospitalization, N (%)**	**33 (18.4)**
**Died after index hospitalization, 6 months, N (%)**	**17 (11.6)**
**Transplanted, N (%)**	**21 (11.7)**

Abbreviations: IQR, interquartile range; MELD-Na, Model for End-Stage Liver Disease-Sodium score.

### QI pass rates

The final list of QIs measured with their corresponding domains and definitions, as well as the pass rates and the total number of encounters eligible for each QI can be found in Table 2 for the AUD domain and Supplementary Table 1 for the other domains. Each encounter, on average, qualified for 5 (IQR 5–6) QIs and achieved 50% (IQR 40%−67%) of recommended care. The AUD domain had the lowest pass rate on aggregate, with only 19.1% (*n* = 18) of patients receiving pharmacotherapy within 6 months and 35.1% (*n* = 33) receiving behavioral therapy within 6 months. Of patients receiving pharmacotherapy, only baclofen (*n* = 4), acamprosate (*n* = 2), naltrexone (*n* = 1) and gabapentin (*n* = 14) were prescribed. These individual agents were not mutually exclusive of each other—3 patients received a prescription for more than one agentduring the 6-month period. There was considerable variation among the remaining domains—ALD-specific care and inpatient cirrhosis care.

**Table 2:** Quality indicators and pass rates for alcohol-use disorder care.

**Quality Indicator**	**QI Pass Rate (%)**	**Eligible for QI (N)**
**Alcohol-use disorder care**		
If patients survived hospitalization for ALD, then they receive a prescription for AUD pharmacotherapy within 6 months.	19.1	94
If patients survive hospitalization for ALD, then they receive AUD behavioral therapy within 6 months.	35.1	94
Total AUD care pass rate.	27	188

Abbreviations: ALD, alcohol associated liver disease; AUD, alcohol use disorder.

### Association between QIs and outcomes

Receipt of behavioral therapy was the only QI found to be associated with lower 6-month mortality (3% vs. 18%, *p* = 0.05) in bivariate analyses **(**Table 3**)**. It was not, however, associated with 30-day readmissions (15% vs. 31%, *p* = 0.14). We did not find significant associations between adherence to the other seven QIs and 6-month mortality (Supplementary Table 2). We found no statistically significant differences in demographics or clinical characteristics, including disease severity, between patients who received and did not receive behavioral therapy (Supplementary Table 3).

**Table 3:** Association between receipt of AUD QIs and 6-month mortality.

**Quality Indicator**	**Received**% died within 6 months (no./tot no.)	**Did not receive**% died within 6 months (no./tot no.)	**p-value**
**Alcohol-use disorder care**			
If patients survived hospitalization for ALD, then they receive a prescription for AUD pharmacotherapy within 6 months.	5.9 (1/18)	14.6 (11/75)	*p* = 0.44
If patients survive hospitalization for ALD, then they receive AUD behavioral therapy in 6 months.	3 (1/33)	18 (11/61)	*p* = 0.05

Abbreviations: ALD, alcohol associated liver disease; AUD, alcohol use disorder.

**Supplementary Table 1:** . Quality indicators and pass rates for inpatient cirrhosis care and alcohol-associated liver disease care.

**Quality Indicator**	**QI Pass Rate**	**Eligible for QI (N)**
**Inpatient cirrhosis care**		
If patients with ascites are admitted for symptoms related to ascites or encephalopathy, then they receive a diagnostic paracentesis within 24 h.	85	95
If patients have ascitic fluid PMN ≥250, then they receive antibiotics within 24 h.	100	18
If patients with cirrhosis are admitted with or develop GI bleeding as an inpatient, then they receive antibiotics within 24 h.	84.6	39
If patients present with UGIB, then they receive EGD within 24 h of presentation.	51.1	47
**Alcohol-associated liver disease care**		
If patients are admitted with ALD, then they receive a nutrition consult.	86.6	179
If patients are admitted with severe ALD (MELD >21 or DF >32), then they receive corticosteroids unless they have a contraindication.	17.1	111
If patients are admitted with ALD, then they are counseled on abstinence.	84.4	179

Abbreviations: PMN, polymorphonuclear leukocyte; UGIB, upper gastrointestinal bleed; EGD, esophagogastroduodenoscopy; ALD, alcohol associated liver disease; AUD, alcohol use disorder.

**Supplementary Table 2:** **.** Association between receipt of non-AUD QIs and 6-month mortality.

**Quality Indicator**	**Received**% died within 6 months (no./tot no.)	**Did not receive**% died within 6 months (no./tot no.)	**p-value**
**Inpatient cirrhosis care**			
If patients with ascites are admitted for symptoms related to ascites or encephalopathy, then they receive a diagnostic paracentesis within 24 h.	33.3 (27/81)	21.4 (3/14)	*p* = 0.53
If patients have ascitic fluid PMN ≥250, then they receive antibiotics within 24 h.	n/a	n/a	n/a
If patients with cirrhosis are admitted with or develop GI bleeding as an inpatient, then they receive antibiotics within 24 h.	21.2 (7/33)	33.3 (2/6)	*p* = 0.6
If patients present with UGIB, then they receive EGD within 24 h of presentation.	25 (6/24)	17.4 (4/23)	*p* = 0.72
**Alcohol-associated liver disease care**			
If patients are admitted with ALD, then they receive a nutrition consult.	27.7 (43/155)	16.7 (4/24)	*p* = 0.32
If patients are admitted with severe ALD (MELD >21 or DF >32), then they receive corticosteroids unless they have a contraindication.	36.8 (7/19)	29.3 (27/92)	*p* = 0.58
If patients are admitted with ALD, then they are counseled on abstinence.	24.5 (37/151)	35.7 (10/28)	*p* = 0.24

Abbreviations: PMN, polymorphonuclear leukocyte; UGIB, upper gastrointestinal bleed; EGD, esophagogastroduodenoscopy; ALD, alcohol associated liver disease;.

**Supplementary Table 3:** Receipt of behavioral therapy by demographics and disease severity.

**Variable**	**Received behavioral therapy% (n/tot n)**	**p-value**
Female gender	26 (19/73)	0.36
Male gender	19.8 (21/106)	
Hispanic	23 (12/52)	0.98
Non-Hispanic white	22.7 (20/88)	
Non-Hispanic non-white	21.6 (8/37)	
English speaking	23.8 (38/160)	0.37
non-English speaking	11.1 (2/18)	
MELD 0–10	33.3 (2/6)	0.4
MELD 11–20	25.86 (15/58)	
MELD 21–30	23.61 (17/72)	
MELD 31–40	13.95 (6/43)	

## Discussion

In this study, we measured pass rates of evidence based QIs and evaluated the association between QIs with clinical outcomes during inpatient encounters for AH. On average, only 50% of recommended QIs were delivered during each admission. The AUD domain had the lowest aggregate pass rate at 27% with only 18.2% and 35.1% of patients receiving pharmacotherapy and behavioral therapy, respectively. Among the 9 QIs, we found that receipt of behavioral therapy after discharge was associated with lower 6-month mortality.

Firstly, our findings highlight a critical lack of diverse strategies to address AUD among hospitalized adults with ALD. Alcohol cessation is the only intervention that improves long-term outcomes in this population, but brief counseling alone is not sufficient for addressing AUD(Lackner et al., 2017). AUD treatments, which include behavioral therapy (such as motivational interviewing (Smedslund et al., 2011), cognitive behavioral therapy(Magill et al., 2019)) and pharmacotherapy, are effective, evidence-based, and may be even more valuable when combined.(Ray et al., 2020) Pass rates for receiving these treatments, as measured in this cohort of hospitalized patients with alcoholic hepatitis, are very low and similar to pass rates that have been reported in outpatients with and without cirrhosis. (Hepner et al., 2019) (Rogal et al., 2020). By comparison, pass rates for managing other aspects of all-cause cirrhosis for inpatients and outpatients are overall higher (Gonzalez et al., 2021; Kardashian et al., 2020), AUD care should thus be a critical priority for future improvement efforts and will rely on overcoming specific barriers. For providers, these include a lack of time, little training (Im et al., 2020) and poor access to subspecialty care, while patients may face financial issues, insurance issues, and perceived societal stigma of an addiction diagnosis.(Bazzi and Saitz, 2018; Mellinger et al., 2018) The low pass-rates for pharmacotherapy in particular are not surprising, as a prior survey study found that 84% of hepatology providers were less than comfortable prescribing these drugs, with 71% having never prescribed any AUD pharmacotherapeutic agents.(Im et al., 2020) Nearly a fifth of providers expressed significant concern about potential side effects of these agents. Much of this hesitation is due to the lack of FDA-approval for any of these drugs in patients with advanced liver disease. Baclofen, a gamma-aminobutryic acid B-receptor agonist, is the only drug with randomized control trial data to support its use in cirrhosis.(Addolorato et al., 2007) More prospective trials of FDA-approved pharmacotherapies are needed in patients with liver disease. Critically, unlike the medical QIs analyzed in this study, the AUD treatments required a “two-step” process: the provider to prescribe/generate the referral and the patient to fill and/or attend the clinic session. There are barriers between these two steps—access to transportation, insurance issues—that contributed, in part, to the low AUD pass rates. (Mellinger et al., 2018).

Next, we found that receipt of behavioral therapy within 6 months was associated with improved mortality among patients who survived hospitalization. This aligns with results from a prior retrospective study that showed a similar link between use of alcohol rehabilitation services and improved mortality in hospitalized adults with ALD.(Peeraphatdit et al., 2020) We did not, however, observe a significant association between receipt of pharmacotherapy and outcomes, though there was a trend towards decreased mortality. Since we only measured whether patients were prescribed treatment, this finding may reflect poor daily adherence to recommended therapy, which has been previously demonstrated in AUD populations without liver disease.(Dermody et al., 2018) Further research is necessary to establish the efficacy of AUD pharmaco therapies in ALD, as this, outside of baclofen, has largely been unstudied compared to other populations with AUD.(Winder et al., 2015) Alcohol cessation counseling was done frequently (84.4% pass rate) but was not associated with improved outcomes. This may reflect the lack of formal, inpatient addiction services at our medical center and an inability to assess whether these interventions were grounded in current evidence and accepted best practices.

Interestingly, we also did not identify significant disparities in receipt of AUD treatments, even though racial and ethnic disparities in ALD have been observed with respect to health care utilization and mortality in other studies.(May et al., 2016) It is likely, however, that our small sample size made it more challenging to observe significant differences in care. Surprisingly, we found no significant association between receipt of other QIs for recommended inpatient care and patient outcomes.

An important finding is that ICD codes used to identify patients with alcohol-related liver disease did not effectively capture a population of patients that was actively drinking in the past 60 days when confirmed with manual abstraction; in fact, over 45% were excluded since there were no reports of active drinking. This raises significant concerns about using these codes to identify cohorts of patients who may benefit from interventions targeting AUD. To create effective, tailored interventions, we must have more accurate, validated codes to identify active alcohol use. Validation studies for these codes should be undertaken to inform future secondary data set analyses. For instance, alcohol biomarkers are accurate, objective measurements of recent drinking and their role in validating an actively drinking population with ALD should be investigated.

We acknowledge some limitations. We relied on EHR documentation to determine whether there was adequate receipt of AUD outcomes, but it is possible that patients received care through community-based programs or through facilities outside our health system, which may underestimate the total receipt of care. Similarly, a proportion of patients in our sample did not follow-up in our health system following their index hospitalization, which may underestimate our estimates for hospitalization and mortality. We omitted such patients from our analysis of AUD care to ensure completeness of data. Next, our overall sample size was small, which limited our ability to perform regression analyses. We were additionally unable to account for the success or failure of any AUD-related treatment in maintaining abstinence, nor were we able to account for safety data for pharmacotherapy. Some potential confounders were not included, such as other substance use disorders and the severity of underlying medical co-morbidities. Lastly, this observational study occurred before two major developments that may have shifted perceptions on the importance of AUD treatment in our health system: 1) the publication of the AASLD guidelines on ALD(Crabb et al., 2020), 2) the effects of lockdown during the COVID-19 pandemic, which has led to well-publicized increases in rates of harmful drinking (Kim et al., 2020) and 3) the development of a protocol for early transplantation for patients with ALD. We believe, however, that these have created an optimal environment and timeliness for quality improvement initiatives focused on ALD in this population.

Our study had several strengths. Our population was ethnically diverse and indicative of those with ALD across the US. The mortality rates we observed were similar to those seen in prior studies of patients with AH.(Hughes et al., 2018) The study cohort was validated using standardized criteria and manual chart review to ensure all encounters were representative of patients who had active or recent drinking near the time of admission, consistent with known diagnostic criteria for alcoholic hepatitis. We also evaluated patients over a long study period of 21 months, allowing us to ascertain their post-hospital clinical course, their referral and receipt of AUD care and outcomes. Lastly, compared to other studies, we looked at a broad set of practices in the hospital setting to better understand the association between receipt of health services and patient outcomes.

Based on our findings, there are future directions that we would recommend. First, while there has been development of widely accepted, evidence based QIs for patients with cirrhosis (Kanwal et al., 2019) and updated recent ALD guidelines (Crabb et al., 2020), there should be development of QIs that involve more specific metrics for ALD care. This disease process is unique in that it requires highly specialized care for the liver disease and the addiction disorder. ALD-specific QIs are needed to best inform the care of this population going forward. Second, pilot interventions should be developed to improve AUD care in a number of care settings. Progressive solutions, such as a multi-disciplinary ALD clinic, have begun to emerge in the outpatient setting (Mellinger et al., 2021), however the inpatient setting is distinct in that patients with ALD in that it may be the patient's first form of contact with a healthcare system. We hope that our work will drive the creation of unique pilot interventions that can be applied in the inpatient setting for this at-risk population. Identifying patient and provider barriers to improving QIs in the hospital is a critical next step in research. Understanding factors that may influence relapse in these patients is also critical. The presence of psychiatric co-morbidities, severity of AUD, concurrent use of other substances, worse physical health and unemployment are among the considerations that impact relapse rates in this population. (Moos and Moos, 2006; Sliedrecht et al., 2019) Lastly, the development of novel treatments is needed to address the profound hepatic injury that occurs in severe AH.

In conclusion, we studied the quality of care across 3 different domains for hospital encounters involving patients with alcoholic hepatitis in a large, diverse integrated health system. Our goal was to determine specific intervention targets that are drivers of key outcomes—mortality and hospital readmission—to best direct future QI initiatives. The pass rates among the different domains were variable, with receipt of addiction care being the lowest on aggregate. Patients who received behavioral therapy for AUD had lower 6-month mortality, highlighting a key target for future QI. Further research should focus on firmly linking hospitalized patients with ALD to addiction treatments prior to discharge to ensure optimal care to this high risk and vulnerable population.

Financial/Grant Support: Melvin and Bren Simon Gastroenterology Quality Improvement Program

## Author statement

**Adam Winters:** Conceptualization, methodology, writing – original & draft, writing – review & editing, project administration

**Folasade May:** Conceptulization, methodology, writing – review & editing

**Yun Wang:** Data curation, writing review & editing

**Paul Shao:** Data curation, writing review & editing

**Liu Yang:** Data curation, formal analysis

**Arpan Patel:** Conceptualization, methodology, formal analysis, writing—review & editing, supervision, projection administration

## Declaration of competing interest

The authors report no conflicts of interest.
